# A systematic review and meta-analysis of neoadjuvant chemoimmunotherapy in stage III non-small cell lung cancer

**DOI:** 10.1186/s12890-022-02292-5

**Published:** 2022-12-29

**Authors:** Wei Liu, Tiantian Zhang, Qian Zhang, Li Li, Chunhua Xu

**Affiliations:** 1 Department of Respiratory Medicine, The Affiliated Nanjing Brain Hospital of Nanjing Medical University, 215 Guangzhou Road, Nanjing, 210029 Jiangsu Province China; 2 Clinical Center of Nanjing Respiratory Diseases and Imaging, Nanjing, 210029 Jiangsu China

**Keywords:** Neoadjuvant chemoimmunotherapy, Non-small cell lung cancer, Stage III, Safety and efficacy

## Abstract

**Background:**

Stage III non-small cell lung cancer (NSCLC) is a heterogeneous disease with different subtypes, multidisciplinary teams-led management, and a poor prognosis. Currently, the clinical benefits of stage III NSCLC in the neoadjuvant setting are still unclear. We performed a meta-analysis of published data on neoadjuvant chemoimmunotherapy in stage III NSCLC to systematically evaluate its efficacy and safety.

**Methods:**

We searched the databases to identify eligible studies of neoadjuvant chemoimmunotherapy for stage III NSCLC. The primary outcomes mainly included pathological and radiological response outcomes, the feasibility of surgery, and the safety of the regimen. The pathological and radiological response included the rate of major pathologic response (MPR), complete pathologic response (pCR), radiological response outcomes, and R0 resection; The feasibility included the rate of surgical resection, conversion to thoracotomy, surgical complications, pathological downstaging of clinical disease stage. The safety included the incidence of treatment-related adverse events (TRAEs) and severe adverse events (SAEs). R 4.1.3 software was conducted for data analysis, and *p* < 0.05 was considered statistically significant.

**Results:**

Nine trials containing a total of 382 populations were eligible for the meta-analysis, with the pooled surgical resection rate of 90%. Owing to the large heterogeneity of the single-rate meta-analysis, the random effect model was adopted. The estimated pooled prevalence of MPR was 56% (95%CI 0.39–0.72) and of pCR was 39% (95%CI 0.28–0.51). The pooled rate of TRAEs was 65% (95%CI 0.17–0.99) and SAEs was 24% (95%CI 0.05–0.49).

**Conclusion:**

Compared to neoadjuvant chemotherapy or immunotherapy, neoadjuvant chemoimmunotherapy achieved more pathological and radiological relief, and has a high surgical resection rate and low risk of conversion to thoracotomy and surgical complications, with poor tolerance of toxicity but rarely developing life-threatening adverse events. In conclusion, neoadjuvant chemoimmunotherapy is suggested to be beneficial for stage III NSCLC.

## Introduction

In 2022, lung cancer is the leading cause of cancer death in humans, with only 22% of the 5-year relative survival rate. Stage III NSCLC can be viewed as a locally advanced disease that accounts for 20–35% of NSCLC, and the rates for locally advanced increased by 4.5% annually and 3-year relative survival is 31%, median overall survival ranges from 9 to 34 months [1–3]. It is worth that the extent and localization of TNM sub-stage (IIIA, IIIB, IIIC) of stage III NSCLC are so complex and heterogeneous that it needs to involve multidisciplinary approaches [4]. For resectable stage IIIA NSCLC patients, the various treatments are based on disease status, metastasis of lymph nodes, and tumor diameter. For T3 N1 M0 to perform resection, superior sulcus tumors perform concurrent chemoradiotherapy followed by surgical resection and 2 cycles of adjuvant chemotherapy. For N2 disease the standard of care is concurrent chemoradiotherapy, followed by durvalumab for 1 year. For non N2 stage IIIA, the care is surgical resection with adjuvant chemotherapy. For T4 with (mediastinal or main airway involvement), perform surgery or concurrent chemoradiotherapy if not possible. Conversely, unresectable IIIA and stage IIIB-C receive concurrent chemo-radiotherapy and followed by durvalumab for 1 year [5, 6].

Neoadjuvant regimens revolutionized the treatment and increase both locoregional and systemic control, especially for immunotherapy plus chemotherapy. A meta-analysis concluded that neoadjuvant immunotherapy combined with chemotherapy achieved more pathological relief, but TRAEs and postoperative complications were also increased [7]. A meta-analysis in 2022 reported that neoadjuvant immunotherapy or chemoimmunotherapy was safe and effective in stage I–III NSCLC, and compared with neoadjuvant immunotherapy, neoadjuvant chemoimmunotherapy significantly improved the rate of pathological response without increasing SAEs or delaying surgery [8]. In the atezolizumab with chemotherapy trial (NCT02716038) and avelumab plus chemotherapy trial (NCT03480230), both reported superiority of neoadjuvant chemoimmunotherapy in stage IB–IIIA [9, 10]. Neoadjuvant chemoimmunotherapy brings a new era to the application of surgery for NSCLC, however, there were limited trials of neoadjuvant immunochemotherapy, especially for stage III NSCLC. This meta-analysis focused on stage III NSCLC and aimed to collect relevant studies to assess the safety and efficacy of neoadjuvant chemoimmunotherapy, and offer treatment regimen options.

## Methods

### Search strategy and selection criteria

This systematic review and meta-analysis adhered to the Preferred Reporting Items for Systematic Reviews and Meta-analyses (PRISMA) reporting checklist. PubMed, Embase, and Cochrane Library were searched to include neoadjuvant chemoimmunotherapy in stage III non-small cell lung cancer until 7 March 2022. English search term was as follows: (“lung neoplasms” OR “lung cancer”) AND (“preoperative” OR “surgery” OR “resection” OR “lobectomy”) AND (“neoadjuvant therapy” OR “neoadjuvant chemoimmunotherapy” OR “neoadjuvant chemo-immunotherapy”). It was registered in PROSPERO with the registration number CRD42022325531.

Included criteria were according to the PICOS criteria: (a) Patients: the focused patients diagnosed with stage III NSCLC; (b) Intervention: neoadjuvant chemoimmunotherapy before surgery; (c) Comparator: no restriction on whether to set up control groups or intervention measures; (d) Outcomes: the basic characteristics of the included population and the primary endpoints such as MPR, pCR, surgical resection rate, and adverse reactions, etc. (e) Study design: randomized controlled trials (RCTs), non-randomized controlled trials (non-RCTs), cohort studies, conference abstracts of clinical trials that clearly described the primary outcome. Exclusion criteria were as follows: (a) treatment with immunotherapy alone or immunotherapy combined with other therapies other than chemotherapy; (b) studies that did not report the efficiency and safety. (c) the population including stage I–II NSCLC. (d) duplicate article. The literature search and data extraction were conducted by two reviewers, with disagreements resolved by the consensus.

### Data extraction

The following data were extracted from each eligible study: (I) The characteristics of articles (author, years of publication, NCT number, study phase, and study design). (II) Patient characteristics (simple size, median age, proportion of males, proportion of squamous-cell carcinoma). (III) Neoadjuvant treatment regimen (the cycle of neoadjuvant therapy, the time to perform surgery). (IV) Endpoints: (a) Pathological and radiological indicators: MPR, pCR, radiological response outcomes, and R0 resection; (b) Surgery: resection rate, surgical delay rate, conversion to thoracotomy rate, the incidence of surgical complications, and clinical to pathological downstaging after neoadjuvant therapy; (c) Adverse reactions: incidence of TRAEs and SAEs.

### Statistical analysis

Single rate meta-analysis is an original study that provides only sample sizes and the number of events. Meta-analysis was performed utilizing the Metaprop module in R-4.1.3 software. The effect size was all the pooled prevalence proportions with 95% confidence intervals (CIs) [11, 12]. The test of homogenous was considered significant when the *I*^*2*^ statistic was ≥ 50% or the *p* value was ≥ 0.10, then the random-effects model was adopted. Due to the high heterogeneity of the single rate analysis, sensitivity analysis was conducted to exclude each selected study separately to examine the robustness of the pooled results. Publication bias was assessed by funnel plot and Egger’s test and *p* < 0.05 indicated statistically significant.

### Assessments of publication bias and study quality

The partial MINORS tool was used to evaluate the study quality. The final version of MINORS contained 12 items, the first eight being specifically for non-RCTs: (1) A clearly stated aim; (2) Inclusion of consecutive patients; (3) Prospective collection of data; (4) Endpoint appropriate to the study aim; (5) Unbiased assessment of endpoints; (6) Follow-up period appropriate to the major endpoint; (7) Loss to follow up not exceeding 5%; (8) Prospective calculation of the study size. The items are scored 0 (not reported), 1 (reported but inadequate), or 2 (reported and adequate) [13]. The result is displayed in Table 1. For cohort studies, the Newcastle–Ottawa Scale was used including eight items about selection, comparability, and outcome.

**Table 1:** Assessment of non-randomized controlled trials in the version of MINORS.

Study	A clearly stated aim	Inclusion of consecutive patients	Prospective collection of data	Endpoint appropriate to the study aim	Unbiased assessment of endpoints	Follow-up period appropriate to the major endpoint	Loss to follow up not exceeding 5%	Prospective calculation of the study size	Total score
Rothschild [14]	2	2	2	2	0	1	2	1	12
Provencio [15]	2	2	2	2	0	1	2	1	12
Zhang [16]	2	2	2	2	0	1	2	0	11
Sun [17]	2	2	2	2	0	1	2	1	12
Wang [18]	2	2	2	2	0	1	2	0	11
Zhao [19]	2	2	2	2	0	1	2	1	12

## Results

### **The characteristics of studies**

**Fig. 1:**
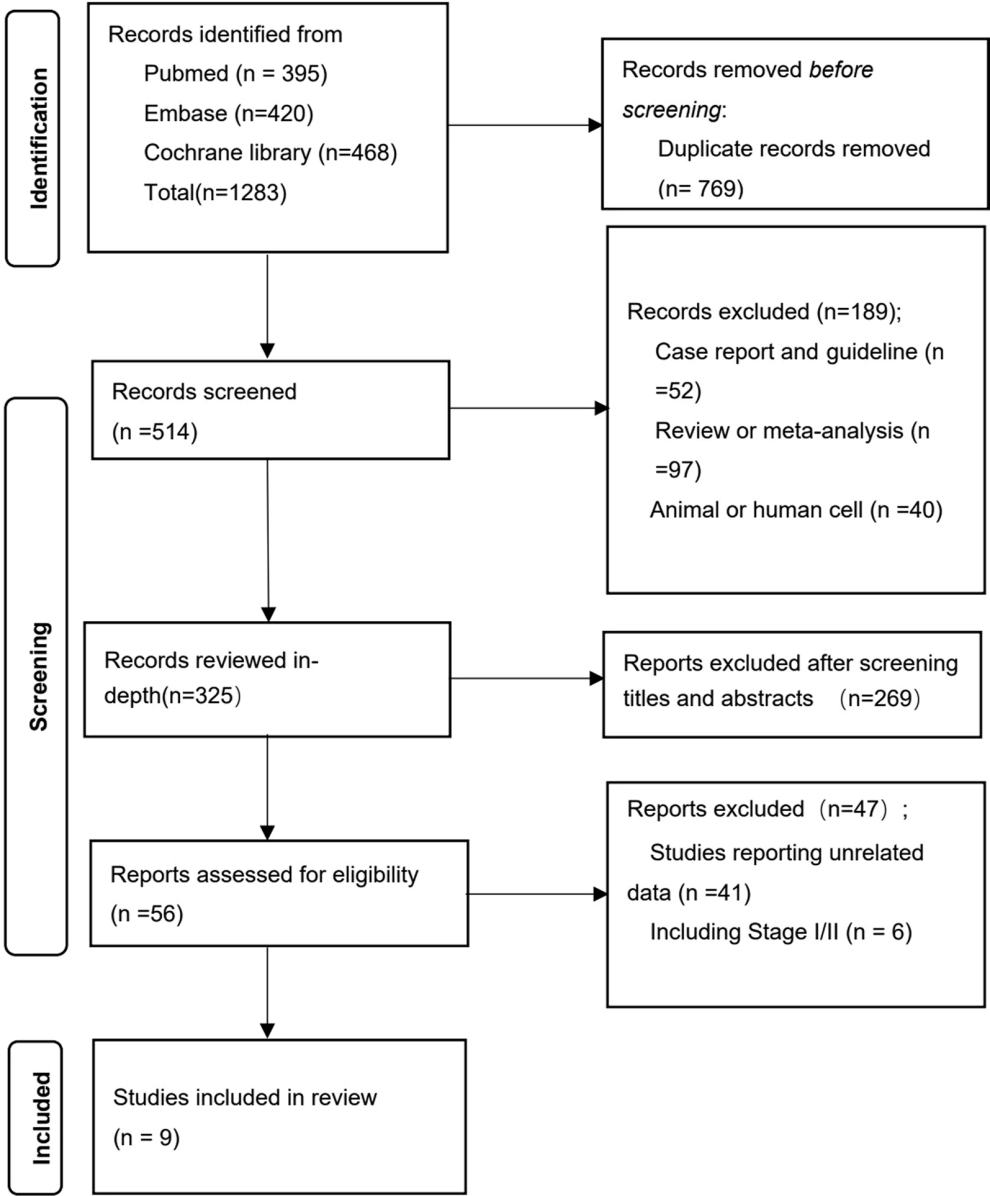
The PRISMA flowchart: the selection process for the eligible studies

The flowchart is summarized in Fig. 1. After removing duplicate and unqualified articles, 9 studies were finally included. Among all the single-arm studies, 6 studies were prospective non-RCTs with phase 2 [14–19] and 3 studies were cohort studies [20–22]. This meta-analysis included different immune checkpoint inhibitors such as nivolumab, sintilimab, pembrolizumab, toripalimab, durvalumab. In clinical trials, the cycle of neoadjuvant therapy was between 2 and 3 cycles. The time to proceed with surgery was between 4 and 7 weeks after neoadjuvant chemoimmunotherapy. Moreover, adjuvant therapy was scheduled to commence on average 1–2 months after surgery and the adjuvant treatment was continued for 12 months. The characteristics of included studies are presented in Table 2.

**Table 2:** Studies characteristics of neoadjuvant chemoimmunotherapy in stage III NSCLC

Study	Stage	NCT number	Study phase	Study design	Neoadjuvant treatment regimen	Neoadjuvant cycle	The time to perform surgery (days)	The time from operation to adjuvant therapy (days)	The time of adjuvant therapy (year)
Rothschild [14]	IIIA(N2)	NCT02572843	2	Multicentre, single-arm	Cisplatin + docetaxel + durvalumab	3	NR	NR	1
Provencio [15]	IIIA(N2)	NCT03081689	2	Multicentre, single-arm	Paclitaxel + carboplatin + nivolumab	3	42–49	21–56	1
Zhang [16]	IIIA	ChiCTR1900023758	2	Single-center, single-arm	Sintilimab + carboplatin + gemcitabine/pemetrexed	2–4	42–49	NR	NR
Sun [17]	IIIA/IIIB	NCT04326153	2	Single-center, single-arm	Sintilimab + nabpaclitaxel + carboplatin	2–3	30–45	28–60	1
Wang [18]	IIIA	NR	NR	Single-centre, single-arm	Nivolumab/pembrolizumab/camrelizumab + Albumin paclitaxel + Carboplatin	2	21–35	NR	NR
Zhao [19]	IIIA or T3-4N2 IIIB	NCT04304248	2	Single-centre, single-arm	Toripalimab + carboplatin + pemetrexed/nab-paclitaxel	3	28–35	28–56	1
Zhai [20]	IIIA/IIIB	NR	NR	Retrospective study	Nivolumab + paclitaxel + carboplatin	3	28–42	NR	(73.4%) patients received at least one cycle of adjuvant nivolumab
Chen [21]	IIIA/IIIB	NR	NR	Retrospective study	Nivolumab/pembrolizumab + carboplatin with paclitaxel	4 (pembrolizumab)2 (nivolumab)	28 (range 4–52)	26	1
Chen [22]	IIIA/IIIB	NR	NR	Retrospective study	Pembrolizumab + chemotherapy	2	33.4 (range 28–35)	NR	NR

*NR* not reported

The meta-analysis enrolled 382 patients in stage III NSCLC, and 336 patients underwent surgery. The median age was 62 years old. The Proportion of males was 56.5–91.7%, and squamous cell carcinoma ranged from 33 to 91.7%. Seven studies reported the rate of surgical delay with no patients experiencing a delay in surgery. The efficacy, safety, and feasibility of neoadjuvant chemoimmunotherapy are shown in Table 3.

**Table 3:** The efficacy, safety and feasibility of neoadjuvant chemoimmunotherapy in stage III NSCLC.

Study	Simple size	Patients with resection	Median age (years)	Proportion of male (%)	Proportion of SCC (%)	Surgical resection rate	R0 resction	MPR	pCR
Rothschild [14]	68	55	NR	NR	NR	81% (55/68)	93% (51/55)	62% (34/55)	18% (10/55)
Provencio [15]	46	41	63 (58–70)	74% (34/46)	35% (16/46)	89% (41/46)	NR	83% (34/41)	63% (26/41)
Zhang [16]	50	30	64.84 ± 9.61	88% (44/50)	56% (28/50)	60% (30/50)	100% (30/30)	43% (13/30)	20% (6/30)
Sun [17]	20	16	59.5 (34–71)	90% (18/20)	80% (16/20)	80% (16/20)	100% (16/16)	63% (10/16)	31% (5/16)
Wang [18]	72	72	62.2 (42–76)	92% (66/72)	92% (66/72)	100% (72/72)	NR	NR	29% (21/72)
Zhai [20]	46	45	63 (56–73)	57% (26/46)	59% (27/46)	98% (45/46)	96% (43/45)	18% (8/45)	53% (24/45)
Zhao [19]	33	30	61 (56–66)	18% (6/33)	55% (18/33)	91% (30/33)	97% (29/30)	67% (20/30)	50% (15/30)
Chen [21]	12	12	61.00 (55.25–66.75)	75% (9/12)	33% (4/12)	75% (9/12)	100% (12/12)	33% (4/12)	42% (5/12)
Chen [22]	35	35	62.17 ± 5.99 (43–72)	83% (29/35)	75% (26/35)	100% (35/35)	100% (35/35)	75% (26/35)	51% (18/35)

Study	Incidence of TRAEs	Incidence of SAEs	Incidence of surgical complications	Surgical delay rate	Convertion to thoracotomy rate	Radiological response outcomes	Pathological downstage
Rothschild [14]	NR	87% (59/68)	NR	NR	NR	58% (39/68)	67% (37/55)
Provencio [15]	93% (43/46)	30% (14/46)	29% (12/41)	0	4/41	76% (35/46)	90% (37/41)
Zhang [16]	90% (45/50)	8% (4/50)	3% (1/30)	0	NR	46% (23/50)	77% (23/30)
Sun [17]	70% (14/20)	35% (7/20)	NR	0	0/16	NR	69% (11/16)
Wang [18]	NR	4% (3/72)	NR	0	NR	NR	NR
Zhai [20]	NR	20% (9/46)	0% (0/45)	NR	NR	61% (28/46)	NR
Zhao [19]	NR	NR	NR	0	20% (6/30)	88% (29/33)	80% (24/30)
Chen [21]	NR	NR	25% (3/12)	0	NR	NR	92% (11/12)
Chen [22]	3% (1/35)	3% (1/35)	0%(0/35)	0	NR	NR	NR

*SCC* squamous cell carcinoma, *MPR* major pathologic response, *pCR* complete pathologic response, *TRAE* treatment-related adverse events, *SAEs* severe adverse events, *NR* not reported

### The efficacy, feasibility, and safety of neoadjuvant chemoimmunotherapy

**Fig. 2:**
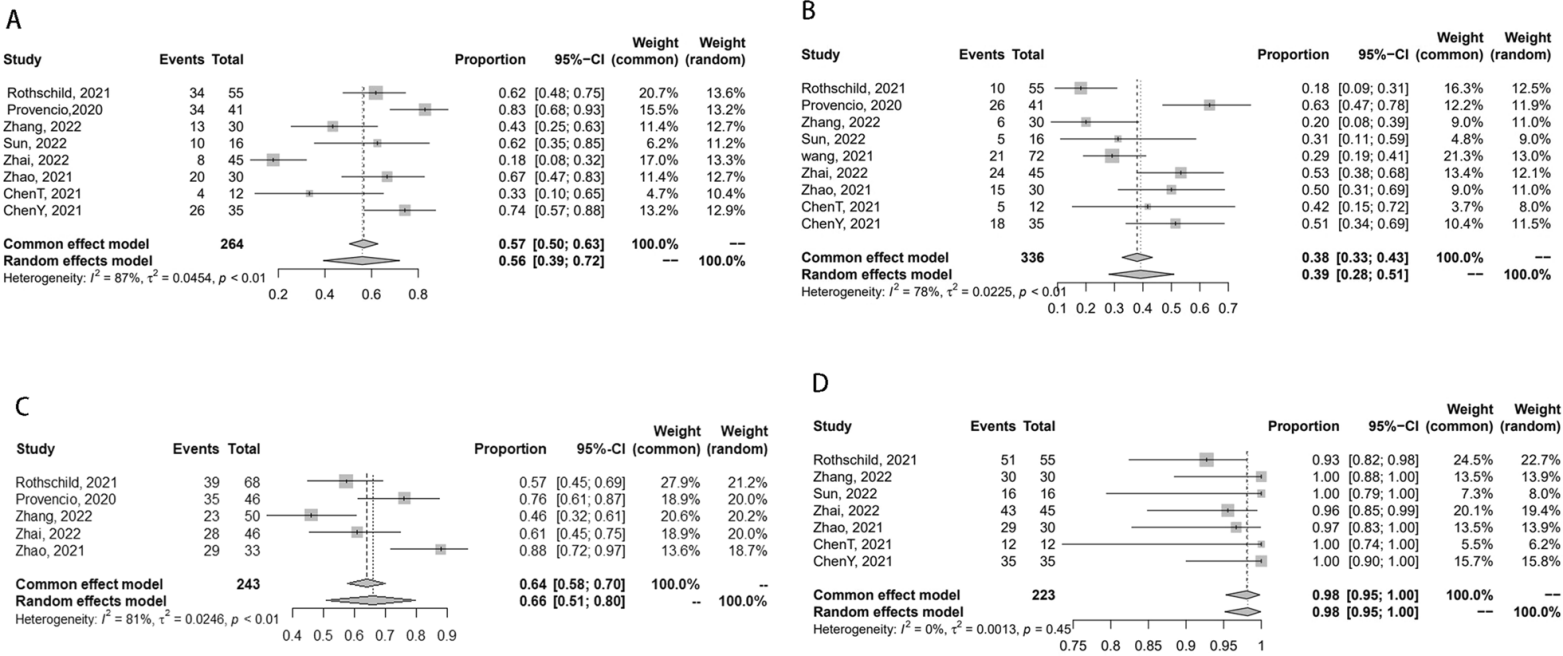
Forest plots of the radiological and pathological response outcomes. **A** The pooled prevalence of MPR; **B** the pooled pCR rate; **C** the pooled prevalence of radiological response outcomes; **D** the occurrence of R0 resection;

Forest plots of the radiological and pathological response outcomes are shown in Fig. 2. MPR is defined as ≤ 10% of viable tumors. The pCR is lack of any viable tumor cells which would be staged as ypT0N0 [23, 24]. The eight studies reported the date of MPR [14–17, 19–22]. Among the 264 patients enrolled, 149 (56%) achieved an MPR. The MPR ranged from 18 to 83%, and generated the pooled prevalence of 56% (95%CI [0.39–0.72], *I*^2^ = 87%, *p <* 0.01) (Fig. 2A). Among the 336 patients enrolled, 130 (39%) achieved an pCR. All eligible studies [14–22] reported the pooled pCR rate was 39% (95%CI [0.28–0.51], *I*^2^ = 78%, *P <* 0.01), which is between 18 and 63% (Fig. 2B). Radiological response outcome was defined as a complete response (CR) or partial response (PR) according to Response Evaluation Criteria in Solid Tumors (RECIST) by CT. The pooled prevalence of radiological response outcomes was 66% (95%CI [0.51–0.80], *I*^2^ = 81%, *P <* 0.01), ranging from 46 to 88% [14–16, 19, 20] (Fig. 2C). R0 resection represents no residual tumor. The occurrence of R0 resection varied from 93 to 100%, with pooled rate of 98% (95%CI [0.95–1.00], *I*^2^ = 0%, *P <* 0.45) [14, 16, 17, 19–22] (Fig. 2D).

**Fig. 3:**
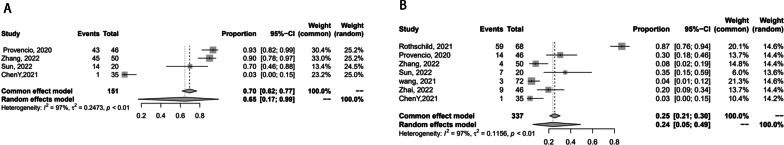
Forest plots of the safety of surgery. **A** The pooled TRAEs rate; **B** the SAE rate

Figure 3 presents the safety of neoadjuvant chemoimmunotherapy. TRAEs were assessed by Common Terminology Criteria for Adverse Events (CTCAE). Only four of the clinical studies provided the incidence of TRAE [15–17, 22]. The pooled TRAEs rate was 65% (95%CI [0.17–0.99], *I*^2^ = 97%, *P <* 0.01), ranged from 3 to 93% (Fig. 3A). SAEs were defined as the grade ≥ 3 TRAEs. Seven studies reported the results of SAEs [14–18, 20, 22]. A total of 337 patients enrolled, and 97 (29%) of them experienced SAEs. The pooled rate of SAEs ranged from 3 to 87% with a pooled incidence of 24% (95%CI [0.05–0.49], *I*^2^ = 97%, *P <* 0.01) (Fig. 3B).

**Fig. 4:**
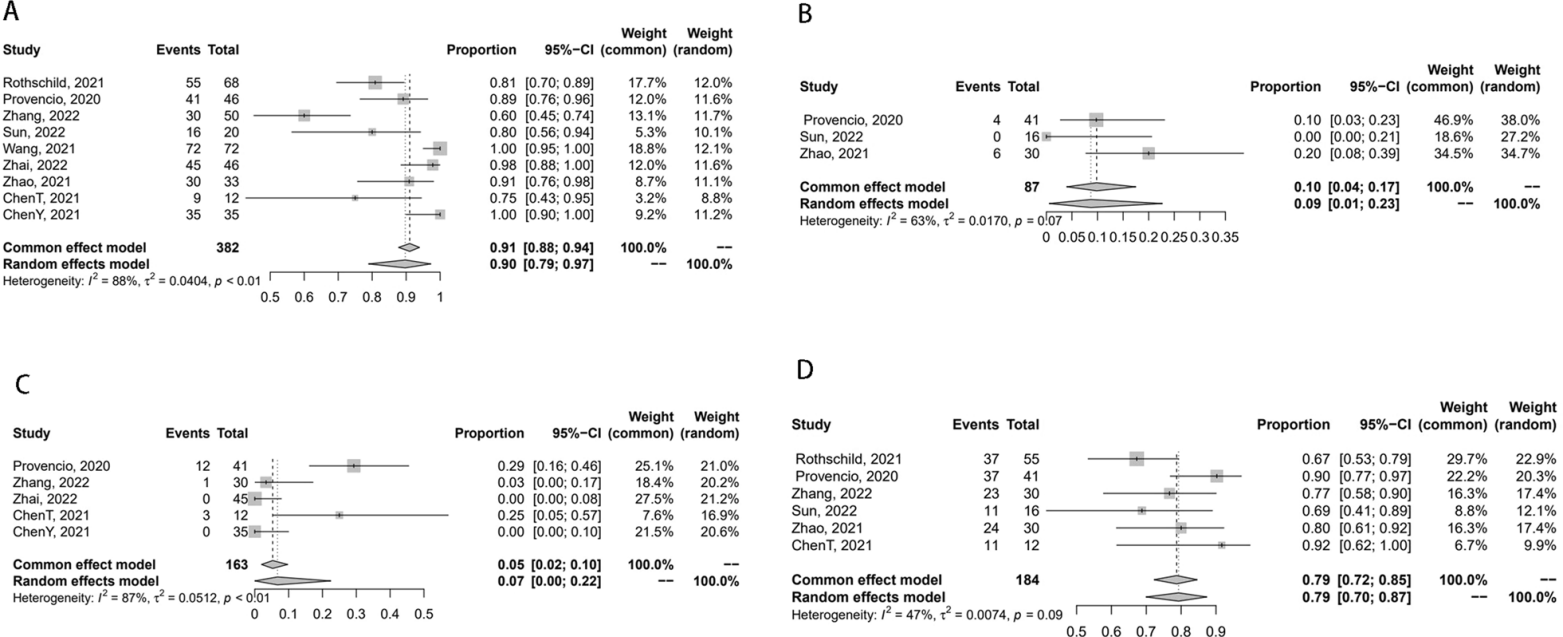
Forest plots of the feasibility. **A** The pooled incidence of surgical resection; **B** the pooled conversion to thoracotomy rate; **C** the rate of surgical complications; **D** the tumor downstaging rate

The meta-analyzed forest plots of feasibility are shown in Fig. 4. A total of 382 patients were enrolled, of which 333 (87%) underwent surgery. The pooled incidence of surgical resection was 90% (95%CI [0.79–0.97], *I*^2^ = 88%, *P <* 0.01), which was between 60 and 100% [14–22] (Fig. 4A). The pooled of conversion to thoracotomy rate was 9% (95%CI [0.01–0.23], *I*^2^ = 88%, *P <* 0.01), which was deemed feasible [15, 17, 19] (Fig. 4B). Surgical complications were observed in 16 of 163 patients who underwent surgery. The pooled incidence of surgical complications was reported 7% (95%CI [0.00–0.22], *I*^2^ = 87%, *P <* 0.01), ranged from 0 to 29% [15, 16, 20–22] (Fig. 4C). Moreover, 143 of the 184 patients (78%) achieved pathological downstaging of the clinical disease stage. The overall tumor downstaging was 79% (95%CI [0.70–0.87], *I*^2^ = 47%, *P =* 0.09), ranged from 67 to 92% [14–17, 19, 21] (Fig. 4D).

### Publication bias and sensitivity analysis

Because of the high heterogeneity, the random effect model was conducted for all the above outcomes, thus phase 3 and large-scale RCTs should be performed to assess the neoadjuvant regimens on the prognosis of stage III NSCLC in the future. The funnel plot was symmetrically distributed and Egger’s test showed that *P* > 0.05, with no obvious publication bias. Most of the results were robust, except for the results of TRAEs and SAEs rate. When we excluded Chen [22], we found that the *I*^2^ of TRAEs was from 97% reduced to 65.4%. When we excluded Rothschild [14], we found that the *I*^2^ of SAEs was from 97% reduced to 82.2%.

## Discussion

The data showed the feasibility of surgery resection, encouraging radiological and pathological response and acceptable adverse reactions in neoadjuvant chemoimmunotherapy. Depending on the results, neoadjuvant immunotherapy plus chemotherapy may be standard care for localized NSCLC.

A study concluded that pCR rate ranged from 0 to 10.5% in phase III neoadjuvant chemotherapy clinical trials, which was less effective than neoadjuvant chemoimmunotherapy [25]. Jia et al. reported the efficacy and safety of neoadjuvant immunotherapy. Compared with the results of neoadjuvant chemoimmunotherapy in our meta-analysis, neoadjuvant immunotherapy has less rate of MPR (37%) and pCR (14%), a similar surgical resection rate (88%), a higher incidence of surgical complications (29%), and surgical delay rate (3%), but good tolerance of toxicity (TRAE16%) [26], this similar conclusion was also suggested in Palmero et al. [27]. Results of CheckMate 816, a phase III trial, reported that the pCR was 23% and event-free survival was 31.6% in stage IIIA NSCLC, moreover, the greater benefit was seen in stage IIIA than in those with stage IB or II patients, which strongly supported our research [28]. Neoadjuvant immunochemotherapy is effective and feasible, it is surprising that increases opportunities for surgery in patients with stage III NSCLC. Therefore, patients diagnosed with stage III may be suitable candidates for neoadjuvant immunochemotherapy. However, the combination of chemotherapy and immunotherapy raised the concern of clinicians on toxicity and maybe increase adverse reactions, especially for patients with poor performance status, which should deserve our attention in the real world [29, 30]. Furthermore, adverse events due to combination of chemotherapy and immunotherapy have been reported to be more common in elderly non-small cell lung cancer patients [31, 32].

The neoadjuvant chemoimmunotherapy could produce the synergistic effect and non-overlapping toxicity, it offered the rationale hypothesis that the antigen load of the intact tumor might enhance a greater breadth of T-cell responses and long-term immunologic memory to reduce the immunosuppressive tumor microenvironment, leading to the ability to assess on-­treatment response, reduce the tumor bulk preoperatively, inhibit the tumor recurrence and enhance tolerability in the preoperative setting [33–36]. The recommendation was made by the European Society of Medical Oncology (ESMO) to initiate durvalumab therapy in PD-L1 ≥ 1% only [37, 38]. High PD-L1 and tumor mutational burden (TMB) responds positively to immunotherapy, those certain patients may be appropriate candidates, so how according to those biomarkers to conduct the regime remains an issue.

To our knowledge, this is the first article to focus on the outcomes of chemoimmunotherapy in the neoadjuvant setting specifically for stage III NSCLC, which greatly decreases the confounding factors of different treatment regimens and populations, and provides a reference for clinicians in the subsequent care of stage III NSCLC.

There are still some limitations in our study. Firstly, the pseudoprogression immune flare may be considered when incorporating immunotherapy into surgical treatment which brings challenges for radiological and pathological assessment. Secondly, there are relatively few and limited sample sizes, and several chemotherapeutic regimens and several types of ICIs were involved, so it makes heterogeneity and difficult to conduct subgroup analysis. Thirdly, the meta-analysis included several retrospective studies and single-center studies, and the stability of results in a single-arm group was different from studies with two groups, with *I*^2^ ≥ 50%. Therefore, it is difficult to directly conclude the *P* value and restrict the reliability of results. Lastly, the study was not able to evaluate the long-term survival outcome of patients who received neoadjuvant chemoimmunotherapy. Phase III trials and comparative study of RCTs in neoadjuvant chemoimmunotherapy are awaited in the future.

## Data Availability

All data generated or analyzed during this study are included in this published article. The data could be freely available for anyone interested.
